# Discovery of Novel Bacterial Cell-Penetrating Phylloseptins in Defensive Skin Secretions of the South American Hylid Frogs, *Phyllomedusa duellmani* and *Phyllomedusa coelestis*

**DOI:** 10.3390/toxins8090255

**Published:** 2016-08-31

**Authors:** Nan Yang, Lei Li, Di Wu, Yitian Gao, Xinping Xi, Mei Zhou, Lei Wang, Tianbao Chen, Chris Shaw

**Affiliations:** Natural Drug Discovery Group, School of Pharmacy, Queen’s University, Belfast BT9 7BL, Northern Ireland, UK; nyang01@qub.ac.uk (N.Y.); Lei.Li@qub.ac.uk (L.L.); dwu03@qub.ac.uk (D.W.); ygao07@qub.ac.uk (Y.G.); m.zhou@qub.ac.uk (M.Z.); l.wang@qub.ac.uk (L.W.); t.chen@qub.ac.uk (T.C.); Chris.Shaw@qub.ac.uk (C.S.)

**Keywords:** amphibian, phylloseptin, antimicrobial, peptide, biofilm, membrane permeability

## Abstract

Phylloseptin (PS) peptides, derived from South American hylid frogs (subfamily Phyllomedusinae), have been found to have broad-spectrum antimicrobial activities and relatively low haemolytic activities. Although PS peptides have been identified from several well-known and widely-distributed species of the Phyllomedusinae, there remains merit in their study in additional, more obscure and specialised members of this taxon. Here, we report the discovery of two novel PS peptides, named PS-Du and PS-Co, which were respectively identified for the first time and isolated from the skin secretions of *Phyllomedusa duellmani* and *Phyllomedusa coelestis*. Their encoding cDNAs were cloned, from which it was possible to deduce the entire primary structures of their biosynthetic precursors. Reversed-phase high-performance liquid chromatography (RP-HPLC) and tandem mass spectrometry (MS/MS) analyses were employed to isolate and structurally-characterise respective encoded PS peptides from skin secretions. The peptides had molecular masses of 2049.7 Da (PS-Du) and 1972.8 Da (PS-Co). They shared typical *N*-terminal sequences and *C*-terminal amidation with other known phylloseptins. The two peptides exhibited growth inhibitory activity against *E. coli* (NCTC 10418), as a standard Gram-negative bacterium, *S. aureus* (NCTC 10788), as a standard Gram-positive bacterium and *C. albicans* (NCPF 1467), as a standard pathogenic yeast, all as planktonic cultures. Moreover, both peptides demonstrated the capability of eliminating *S. aureus* biofilm.

## 1. Introduction

Amphibians possess a special innate skin defence system to protect them from being preyed upon by predators and infected by microorganisms in their living environments. So far, hundreds of antimicrobial peptides have been discovered in amphibian skins and these have been widely-studied in recent decades [1,2,3,4]. These amphibian antimicrobial peptides have been divided into different families according to structural similarities and these include the brevinins, esculentins, temporins, dermaseptins, phylloseptins and bombinins [5,6,7,8]. Peptides within a certain family can inhibit the growth of selected bacteria and fungi and some may also possess anti-cancer and/or anti-viral activities [9,10,11].

*Phyllomedusa* is a genus of leaf frogs within the hylid frog subfamily, *Phyllomedusinae* [12]. So far, more than 80 antimicrobial peptides have been discovered from the skin secretions of species within this subfamily and these peptides have been divided into seven peptide families including the dermaseptins, phylloseptins (PS), plasticins, dermatoxins, phylloxins, hyposins, and orphan peptides [13]. The prototype of the phylloseptin (PS) family, was first reported in 2005 [14]. In the past decade, more than 40 novel PS peptides have been identified and all demonstrate a broad-spectrum of antimicrobial activities, especially significantly inhibiting the growth of Gram-positive bacteria and fungi [14,15,16,17,18,19].

*Phyllomedusa duellmani* and *Phyllomedusa coelestis* are members of genus *Phyllomedusa*. Unlike their relatives, *Phyllomedusa hypochondrialis*, *Phyllomedusa sauvagei* and other species, which have been widely studied, only a few bioactive peptides have been reported from these two species. These two species are found in northern Peru and the population status of *Phyllomedusa duellmani* is still shown as data deficient on the Red List of Endangered Species [20].

In this study, “shotgun” cloning was performed using both 3′RACE and 5′RACE polymerase chain reaction (PCR)to obtain full-length nucleotide sequences encoding the open reading frames of their respective PS biosynthetic precursors. The amino acid sequences of predicted PS peptides were confirmed by tandem mass spectrometry (MS/MS) fragmentation using electrospray ion trap mass spectrometry. After chemical synthesis of replicates of both peptides, their biological activities were investigated in antimicrobial and haemolysis bioassays.

## 2. Results

### 2.1. “Shotgun” Cloning of Novel Peptide Precursor-Encoding cDNAs and Bioinformatic Analyses

**Figure 1:**
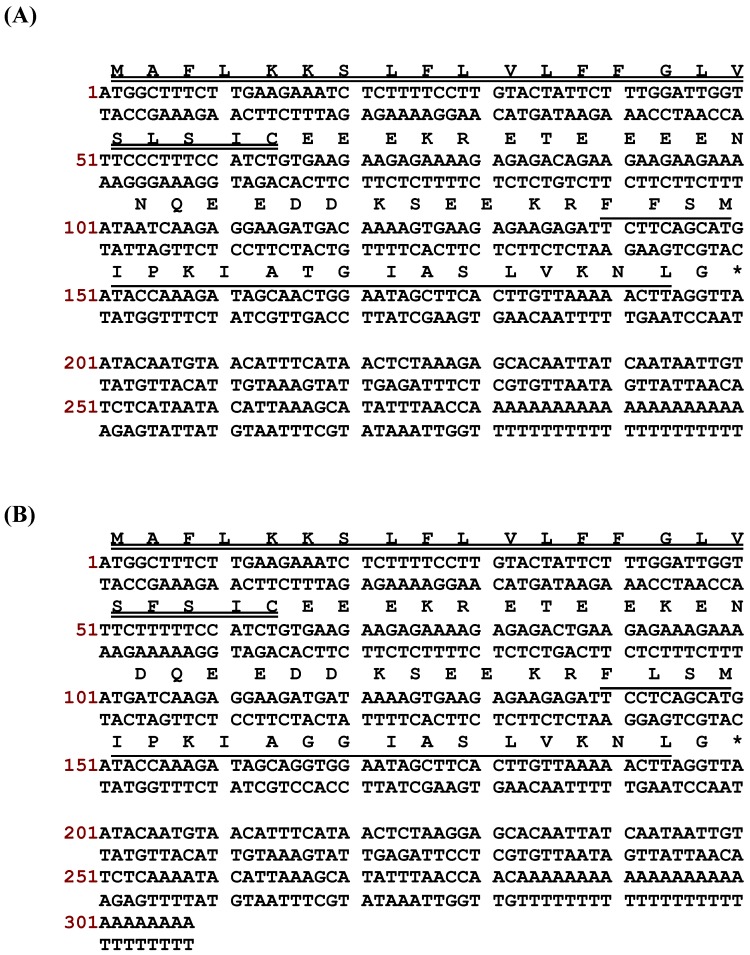
Nucleotide and translated open-reading frame amino acid sequences of cloned cDNAs encoding precursors of novel phylloseptin peptides, PS-Du (**A**) and PS-Co (**B**). Putative signal peptides are double-underlined, mature peptides are single–underlined and stop codons are indicated by asterisks.

Degenerate primers were used for interrogating the skin secretion-derived cDNA libraries of *Phyllomedusa duellmani* and *Phyllomedusa coelestis*. Two full-length cDNAs, encoding PS-Du and PS-Co, were cloned repeatedly (at least 10 clones for each) from the skin secretion-derived cDNA libraries of *Phyllomedusa duellmani* and *Phyllomedusa coelestis*, respectively. They were named PS-Du and PS-Co, respectively, reflecting their species names. The sequences of nucleotides and translated open reading frame amino acids for both peptides are shown in Figure 1A, B. Their structural topology consisted of five typical regions, including a putative signal peptide region of 22 amino acid residues, an acidic “spacer” peptide, typical -KR- propeptide convertase processing sites, a mature peptide of 19 amino acid residues and a Gly residue at the *C*-terminus which acts as an amide donor for providing the post-translational amide modification in each case.

**Figure 2:**
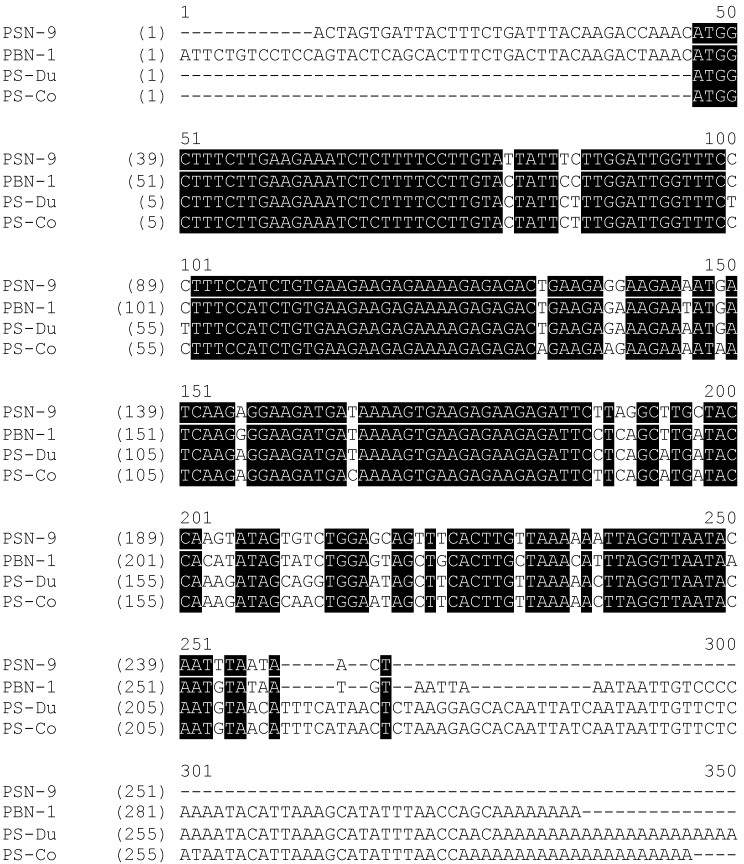
Alignments of the full-length nucleotide sequences of cDNAs encoding four PS precursors, PSN-9 (Accession No. Q0VZ38), PSN-1 (Accession No. Q800R3), PS-Du and PS-Co. Black shading indicates identical sequences between four individual precursors and gaps are inserted to maximize alignments.

**Figure 3:**
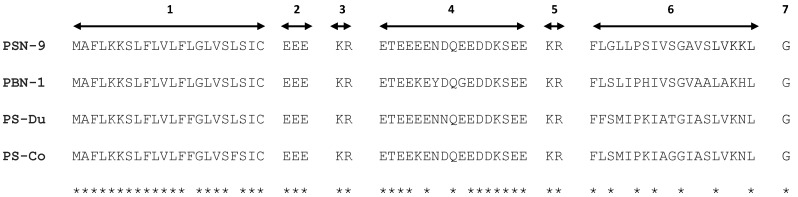
Alignments of cDNA-deduced open-reading frame amino acid sequences of four PS precursors, PSN-9 (Accession No.Q0VZ38), PSN-1 (Accession No.Q800R3), PS-Du and PS-Co, demonstrating 7-regions of topological structures with asterisks indicating sites of amino acid sequence identities. 1—Putative signal peptide; 2 & 4—Acidic amino acid residue-rich spacer peptides; 3 & 5—Dibasic propeptide convertase processing site; 6—Mature PS peptide; 7—Glycine residue amide donor.

Both putative mature peptides were subjected to bioinformatics analysis by use of the National Center for Biotechnology Information (NCBI ) protein Basic Local Alignment Search Tool (BLASTp) program, which found that PS-Du and PS-Co were new phylloseptins. PS-Du and PS-Co showed a high degree of structural identity to phylloseptins from other *Phyllomedusa* frogs, including the well-studied PSN-9 (accession No. Q0VZ38) from *Phyllomedusa hypochondrialis* and PSN-1 (accession No. Q800R3) from *Phyllomedusa bicolor.* The alignment of open-reading frame nucleotide and amino acid sequences of PS-DU, PS-Co, PSN-9 and PSN-1, was established by use of Vector NTI software (Version 11.5, 2010, Life Technologies, Carlsbad, CA, USA), and these are shown in Figure 2 and Figure 3. The nucleotide sequence of both PS-Du and PS-Co precursors, have been deposited in the European Molecular Biology Laboratory (EMBL) Nucleotide Sequence Database under the accession codes LN999522 and LN999523.

The alignments demonstrated a high degree of similarity in both nucleotide and deduced amino acid sequences. More than 85% nucleic acid sequence identities between these four full-length nucleotide sequences were observed, excluding the gaps. This demonstrated highly-conserved genetic information from this subfamily. Meanwhile, the deduced amino acid sequences of these four precursors demonstrated the same topological structures and these are shown in Figure 3.

### 2.2. Fractionation of Skin Secretions, Identification and Structural Characterisation of PS-Du and PS-Co

**Figure 4:**
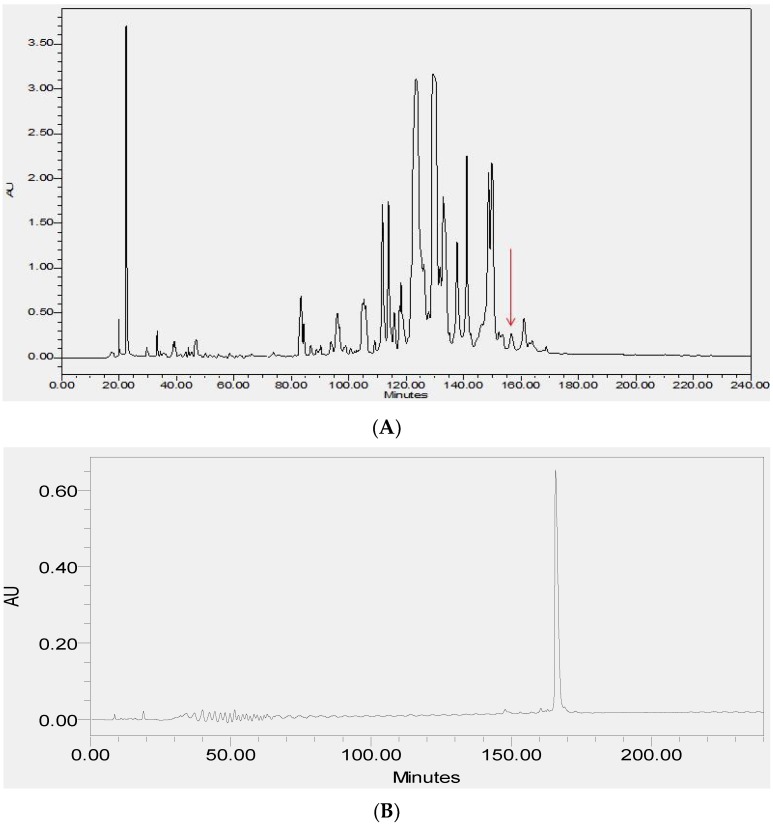

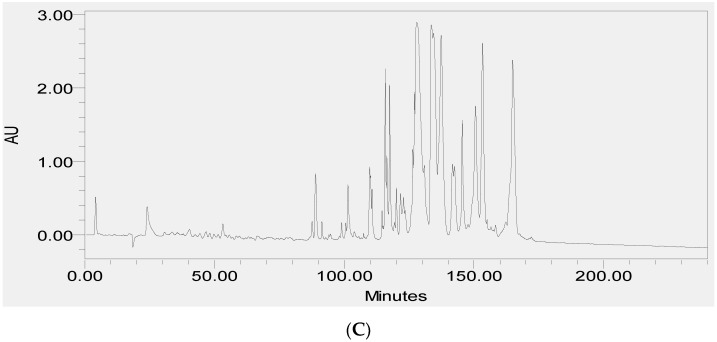
Reverse-phase high-performance liquid chromatography (RP-HPLC) chromatogram of the skin secretion of *Phyllomedusa duellmani* (**A**) with arrow showing the absorbance peak corresponding to natural PS-Du. Reverse phase HPLC profile of synthetic PS-Du (**B**) with absorbance peak at relevant position to that in (**A**); Co-elution reverse phase HPLC chromatogram of synthetic PS-Du added to crude skin secretion of *Phyllomedusa duellmani* (**C**) with absorbance peak at relevant position to that in (**A**). The *Y*-axis shows the relative absorbance in absorbance units at 214 nm and the *X*-axis shows the retention time in minutes.

**Figure 5:**
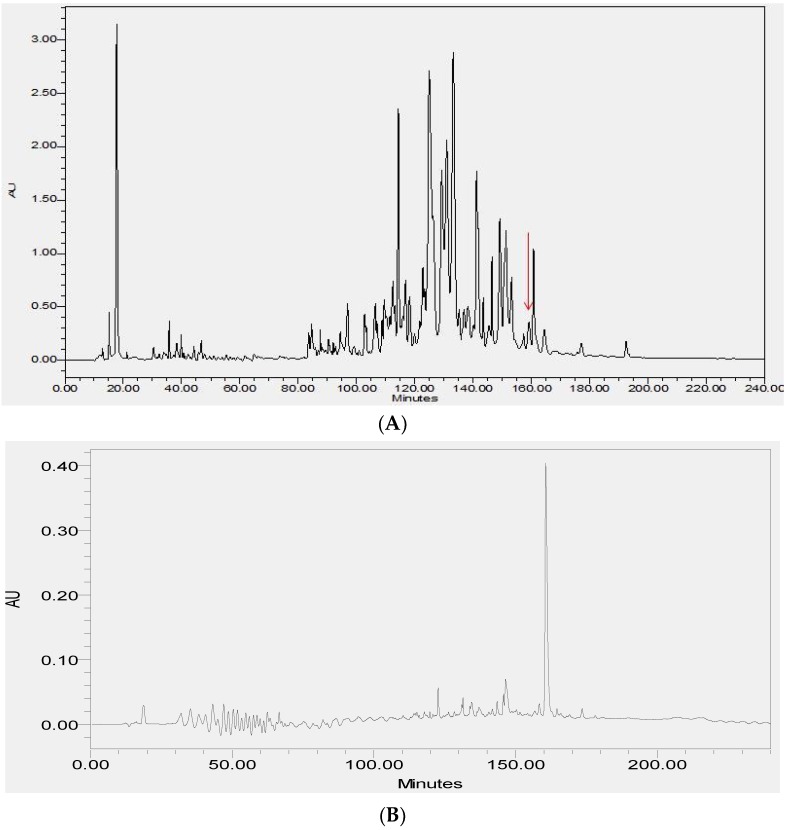

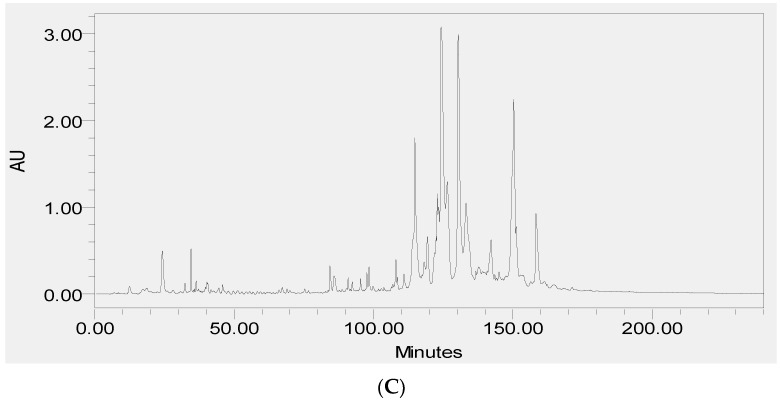
Reverse phase HPLC chromatogram of the skin secretion of *Phyllomedusa coelestis* (**A**) with arrow showing the absorbance peak corresponding to natural PS-Co. Reverse phase HPLC profile of synthetic PS-Co (**B**) with absorbance peak at relevant position to that in (**A**); Co-elution reverse phase HPLC chromatogram of synthetic PS-Co added to crude skin secretion of *Phyllomedusa coelestis* (**C**) with absorbance peak at relevant position to that in (**A**). The *Y*-axis shows the relative absorbance in absorbance units at 214 nm and the *X*-axis shows the retention time in minutes.

**Figure 6:**
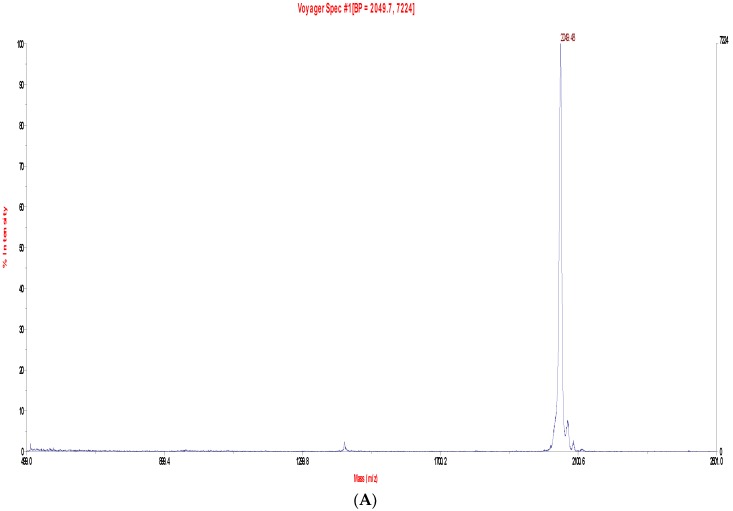

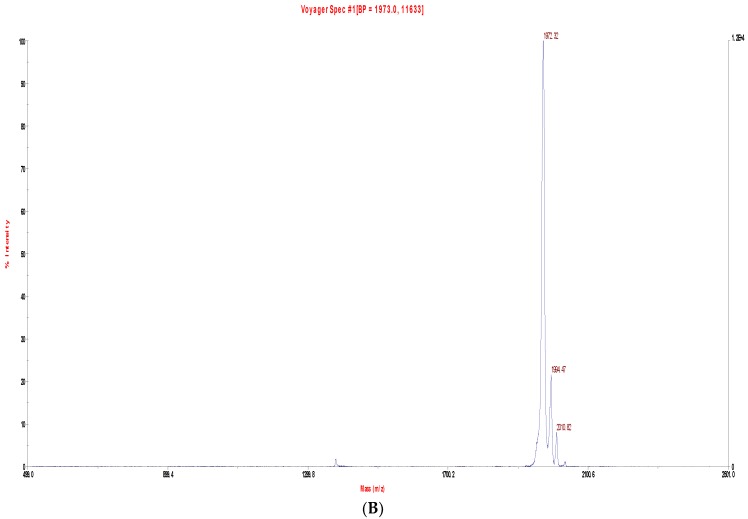
MALDI-TOF (Perceptive Biosystem, Bedford, MA, USA) mass spectra of skin secretion fraction of *Phyllomedusa duellmani* corresponding to PS-Du (**A**); and skin secretion fraction of *Phyllomedusa coelestis* corresponding to PS-Co (**B**).

**Figure 7:**
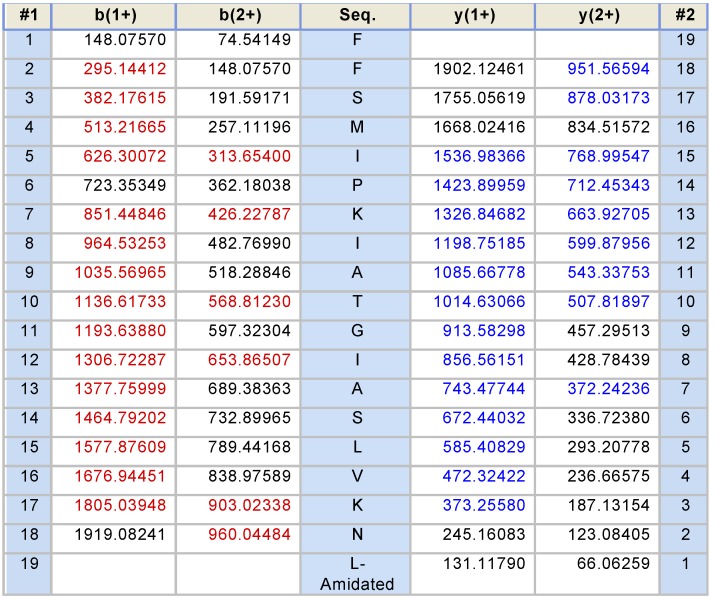

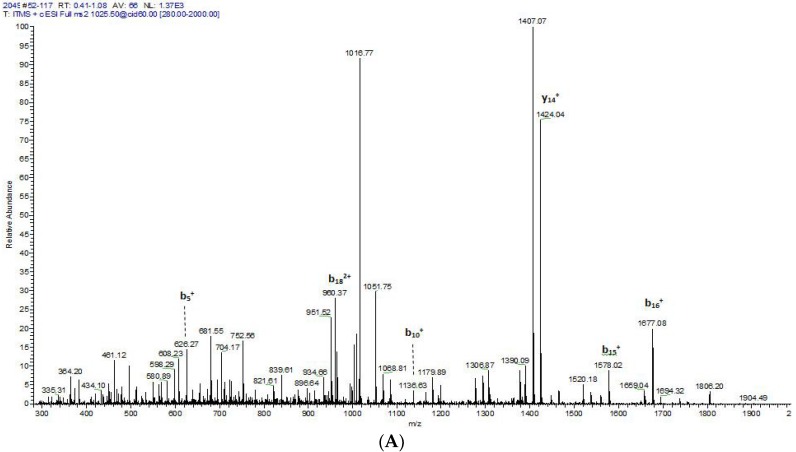

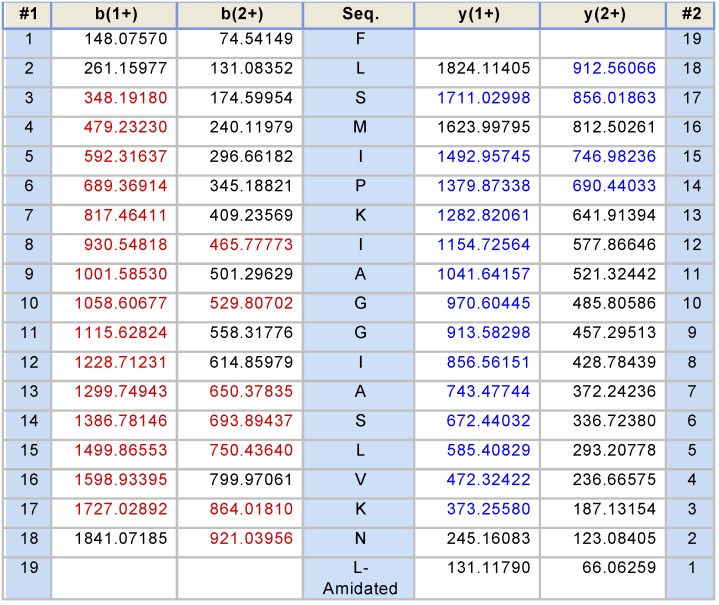

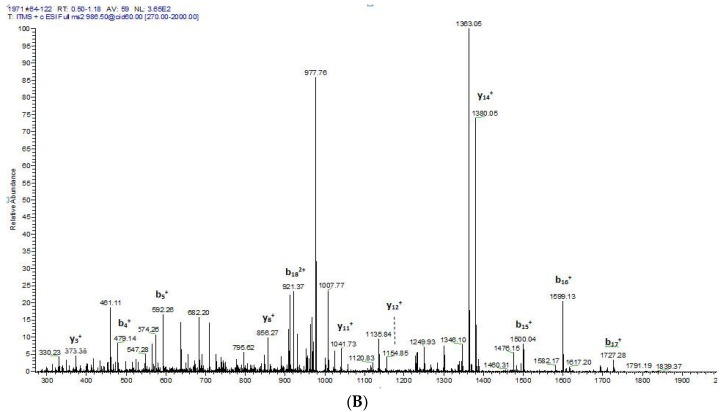
Electrospray ion-trap MS/MSfragmentation datasets and MS/MS fragment scans derived from ions corresponding in molecular mass to PS-Du (**A**) and PS-Co (**B**). Expected singly- and doubly-charged *b*-ions and *y*-ions arising from MS/MS fragmentation were predicted using the MS Product programme available through Protein Prospector on-line. Actual fragment ions observed following MS/MS fragmentation are indicated in blue and red typefaces.

The lyophilized crude skin secretions of *Phyllomedusa duellmani* and *Phyllomedusa coelestis* were respectively fractioned by reversed-phase high-performance liquid chromatography (RP-HPLC) and the chromatograms are shown in Figure 4A and Figure 5A, with arrows indicating the retention times/elution positions of peptides with masses coincident with the approximate predicted molecular masses of PS-Du and PS-Co. The HPLC elution profile of synthetic PS-Du and its co-elution profile with the crude skin secretion of *Phyllomedusa duellmani* is shown in Figure 4B,C. Likewise, the HPLC elution profile of synthetic PS-Co and its co-elution profile with the crude skin secretion of *Phyllomedusa coelestis* is shown in Figure 5B,C. The masses of the peptides in fractions corresponding to PS-Du and PS-Co were detected using matrix-assisted laser desorption/ionization time-of-flight mass spectrometry (MALDI-TOF MS) on a linear time-of-flight Voyager DE mass spectrometer (Perceptive Biosystem, Bedford, MA, USA) (Figure 6). The amino acid sequence of PS-Du and PS-Co were further analysed by MS/MS fragmentation sequencing shown in Figure 7A, B. The amino acid sequences of the mature peptides, PS-Du and PS-Co, were thus unequivocally identified and the glycine (G) residue at the carboxyl terminus of both precursors was also confirmed as an amide donor.

### 2.3. Secondary Structure Prediction of PS-Du and PS-Co.

**Figure 8:**
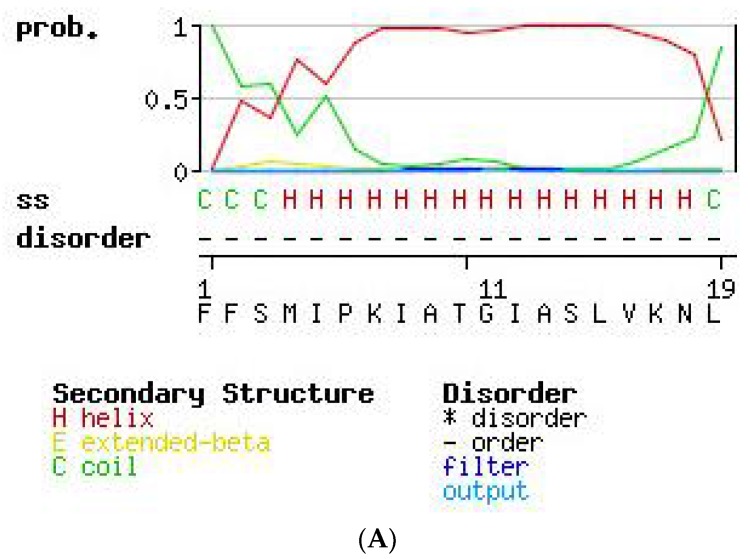

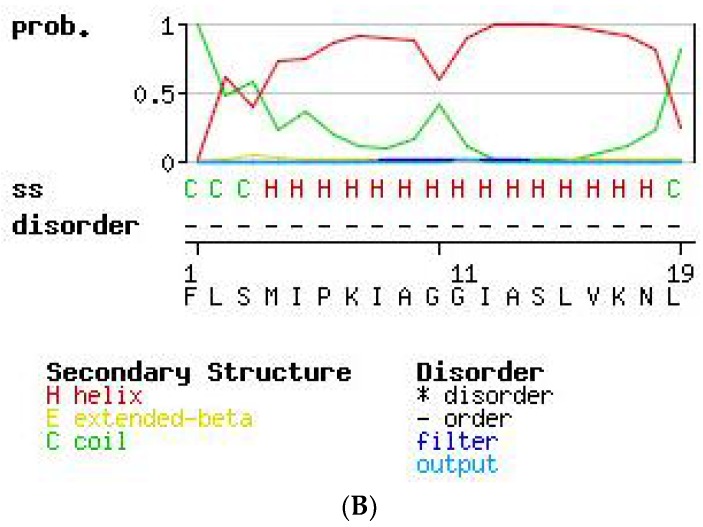
Secondary structure prediction analysis of PS-Du (**A**) and PS-Co (**B**) using SWISS-Model (http://swissmodel.expasy.org) suggesting a large proportion of α-helix in both. The red and green lines indicate the probability of forming regions of helix and coil, respectively.

The secondary structures of PS-Du and PS-Co were predicted through software modeling on the SWISS-Model (http://swissmodel.expasy.org) [21,22,23,24]. The server analysed PS-Du (Figure 8A) and PS-Co (Figure 8B) and found that both peptides contained a large proportion of α-helical domain.

### 2.4. Antimicrobial and Haemolytic Activities of PS-Du and PS-Co and Those of Their Structurally-Modified Analogues, PS-Du K7H, and PS-Co K7H

**Figure 9:**
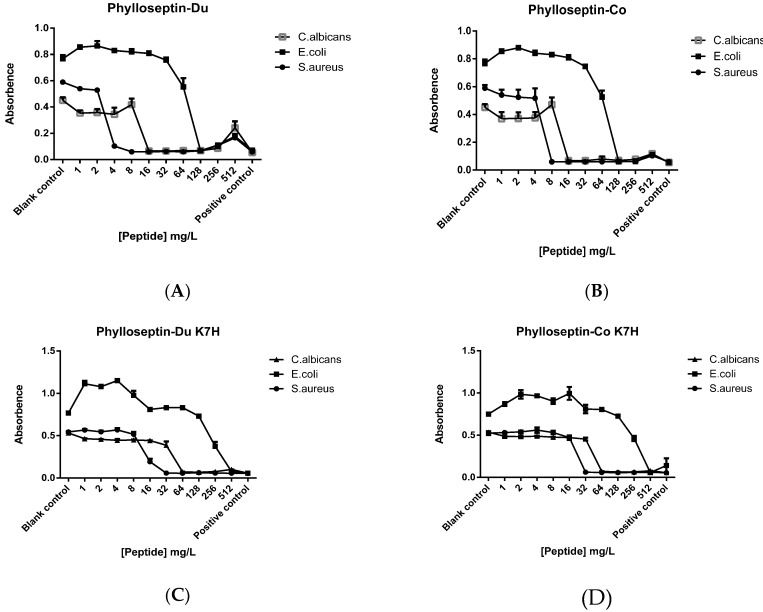
Minimal inhibitory concentration (MIC) curves obtained following incubation of *C. albicans*, *E. coli*, and *S. aureus* with the natural peptides, PS-Du (**A**), PS-Co (**B**) and the structurally-modified peptides, PS-Du K7H (**C**) and PS-Co K7H (**D**) at concentrations ranging from 512 mg/L to 1 mg/L. The blank control was established by culture medium and the positive control was represented by growth culture. Data represent means ± SEM (standard error of the mean) of 5 replicates.

**Figure 10:**
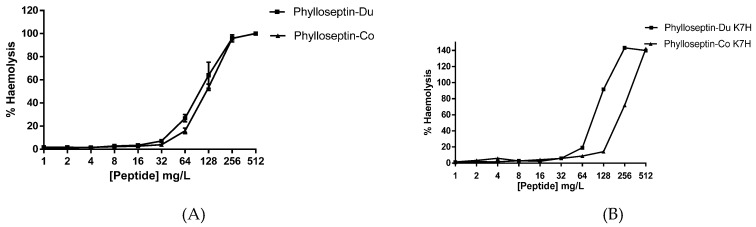
The haemolytic activity of PS-Du, PD-Co (**A**) and PS-Du K7H PS-Co K7H (**B**) at concentrations ranging from 512 mg/L to 1 mg/L. Percentage of haemolysis was evaluated and calculated by comparing values those of the positive control established by using 1% Triton X-100. Data represent means ± SEM of 5 replicates.

Synthetic PS-Du and PS-Co and their respective structurally-modified analogues, PS-Du K7H and PS-Co K7H, exhibited growth inhibitory activity against the Gram-positive bacterium, *S. aureus*, the Gram-negative bacterium, *E. coli* and the potentially-pathogenic yeast, *C. albicans*. MICs (minimal inhibitory concentrations）of all four peptides are summarised in Table 1 and MIC curves are shown in Figure 9. The skin secretion-derived peptides, PS-Du and PS-Co, showed similar potencies with MIC values of 8 mg/L against *S. aureus*, 128 mg/L against *E. coli* and 16 mg/L against *C. albicans*. The modified peptide analogues, PS-Du K7H and PS-Co K7H, showed similar inhibition with MIC values of 32 mg/L towards *S. aureus*, 512 mg/L towards *E. coli* and 64 mg/L towards *C. albicans*. Both natural peptides, PS-Du and PS-Co, and modified peptides, PS-Du K7H and PS-Co K7H, exhibited moderate haemolytic effects on horse red blood cells as shown in Figure 10.

### 2.5. Anti-Biofilm and Cell-Membrane Permeabilization Activities of Natural Peptides, PS-Du and PS-Co

**Figure 11:**
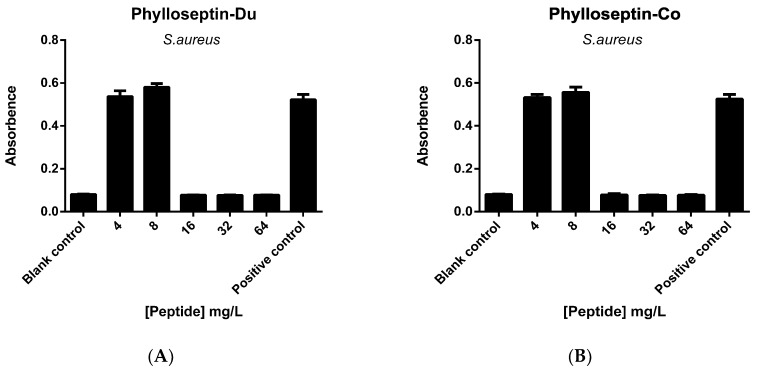
The MBEC (minimal biofilm eradication concentration) of PS-Du (**A**) and PS-Co (**B**) against *S. aureus* biofilm with peptide concentrations ranging from 4 mg/L (0.5 MIC) to 64 mg/L (8 MIC). Blank control was set up with culture medium, and positive control was represented by *S. aureus* biofilm growth culture. Data represent means ± SEM of 9 replicates.

**Figure 12:**
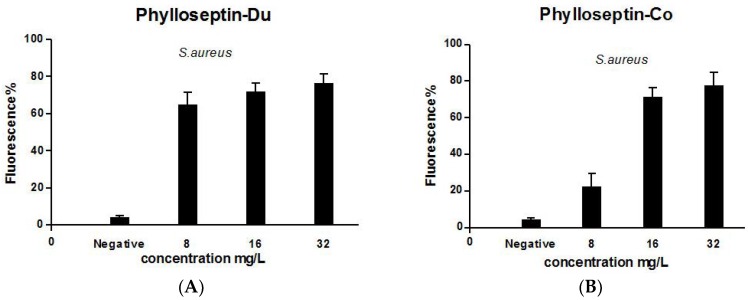
Cell-membrane permeability effects of PS-Du (**A**) and PS-Co (**B**) on *S. aureus* detected by the SYTOX Green (Life technologies, Carlsbad, CA, USA) assay at peptide concentrations corresponding to 1 MIC, 2 MIC and 4 MIC. Positive membrane permeabilization was obtained following incubation of *S. aureus* with 70% isopropyl alcohol. The negative control was represented as vehicle only. Data represent means ± SEM of 5 replicates.

The activities of PS-Du and PS-Co against *S. aureus* biofilm were tested and both peptides possessed biofilm eradication capability with an MBEC (minimal biofilm eradication concentration) of 16 mg/L (Figure 11). Additionally, PS-Du and PS-Co had the capability of cell-membrane permeabilization at concentrations of 8 mg/L and 16 mg/L, respectively (Figure 12). Each assay was carried out over at least three individual experiments with three replicates in each.

## 3. Experimental Section

### 3.1. “Shotgun” Cloning of Novel Phylloseptin Precursor-Encoding cDNAs from Skin Secretion-Derived cDNA Libraries of Phyllomedusa Duellmani and Phyllomedusa Coelestis

Lyophilised skin secretions of *Phyllomedusa duellmani* and *Phyllomedusa coelestis* were obtained from Mr. Juan Chavez, Venom Peru Company (PeruBiotech E.I.R.L, Huánuco, Peru).

The lyophilised skin secretions from both species were separately dissolved in 1 mL of cell lysis/binding buffer (Life technologies, Oslo, Norway). Magnetic oligo-dT beads were used to isolate the polyadenylated mRNA following the procedure described by the manufacturer (Life technologies, Oslo, Norway). To acquire full-length prepropeptide nucleic acid sequence data, a SMART-RACE kit (Clontech, Palo Alto, CA, USA) was employed with a nested universal primer (NUP) (supplied in the kit) and a degenerate primer pool (5′- ACTTTCYGAWTTRYAAGMCCAAABATG-3′ Y = C + T, W = A + T, R = A + G, M = A + C, B = T + C + G) designed to a segment of the 5′-untranslated region of phylloxin cDNA from *Phyllomedusa bicolor* (EMBL Accession No. AJ251876) and the opioid peptide cDNA from *Pachymedusa dacnicolor* EMBL Accession No. AJ005443). The procedure was again as outlined by the manufacturer. The PCR cycling program was as follows: Initial denaturation step: 90 s at 9 4 °C; 35 cycles: denaturation 30 s at 94 °C, primer annealing for 30 s at 58 °C; extension for 180 s at 72 °C. PCR products were analysed by DNA-gel electrophoresis, purified and cloned using a pGEM^®^-T Easy vector system (Promega Corporation, Southampton, UK) and the selected samples were sequenced using an ABI 3100 automated sequencer (Applied Biosystems, Foster City, CA, USA). The Blast Alignment Search Tool (BLAST) of the National Center for Biotechnology Information (NCBI) was used to study the similarities of the novel amino acid sequences with the known sequences in the BLASTp database. Alignments were established to compare the novel sequences with the two identified sequences, PSN-9 (Accession No. Q0VZ38) and PBN-1 (Accession No. Q800R3).

### 3.2. Chromatographic Isolation and Structural Characterisation of the Two Novel Phylloseptins from the Skin Secretions of Phyllomedusa Duellmani and Phyllomedusa Coelestis

Seven mg of lyophilised skin secretions from each species were separately dissolved using 1.25 mL of trifluoroacetic acid (TFA)/water (0.05:99.95, *v*/*v*). The insoluble microparticulates were cleared by centrifugation (2500× *g* for 5 min). The clear supernatants were carefully decanted into a 2 mL screw top vial (Waters, Milford, MA, USA) and placed on an autosampler. The RP-HPLC system used consisted of a Waters 2707 auto sampler, a Waters 1525 HPLC pump and a Waters 2489 UV detector (Waters, USA). The sample solution was separated using a Jupiter C5 reverse phase HPLC column (250 mm × 4.6 mm, Phenomenex, Macclesfield, UK). A linear gradient formed from 0.05/99.95 (*v*/*v*) TFA/water to 0.05/19.95/80.0 (*v*/*v*/*v*) TFA/water/acetonitrile in 240 min at a flow rate of 1 mL/min was employed to elute peptides. Fractions were collected at minute intervals by an automated fraction collector (GE Healthcare, Little Chalfont, UK). Each fraction was subjected to molecular mass analysis by means of a Voyager DE MALDI linear time-of-flight mass spectrometer (Perseptive Biosystems, Bedford, MA, USA) to construct a mass spectral library of skin secretion peptides. The instrument was calibrated in the range of 1–4 kDa and the accuracy of mass determinations was ±0.1%. The computed molecular masses of predicted mature peptides deduced from encoded cDNA were used to interrogate the mass spectral library to identify the putative peptides. The fractions containing the peptides of identical masses to putative novel cDNA-encoded peptides were each subjected to primary structural analysis by MS/MS fragmentation sequencing using an LCQ-Fleet ion-trap mass spectrometer (Thermo Fisher Scientific, San Francisco, CA, USA).

In addition to the primary structure characterisation of the two novel peptides, further -secondary structure prediction was performed using the bioinformatics tool, SWISS-MODEL [21,22,23,24].

### 3.3. Solid-Phase Peptide Synthesis of the Two Novel Peptides and Their Structurally- Modified Analogues

Four peptides were chemically-synthesised by solid phase Fmoc chemistry using a Tribute automated solid-phase peptide synthesiser 4 (Protein Technologies, Tucson, AZ, USA). The amino acid sequences of the two novel frog skin phylloseptin peptides were FFSMIPKIATGIASLVKNL-NH_2_ and FLSMIPKIAGGIASLVKNL-NH_2_, and they were named PS-Du and PS-Co, respectively. The amino acid sequences of the two single-site modified analogues were FFSMIPHIATGIASLVKNL-NH_2_ and FLSMIPHIAGGIASLVKNL-NH_2_. Both contained a His for Lys substitution at position 7, and they were named as PS-Du K7H and PS-Co K7H. All the dry amino acids were weighed and mixed with 2-(1*H*-benzotriazol-1-yl)-1,1,3,3,-tetramethyluronium hexafluorophosphate (HBTU) activtator and transferred to the reaction vessel containing rink amide MBHA resin on the synthesiser. The deprotection of the Fmoc groups was performed in 20% piperidine in dimethylformamide (DMF). The peptide bond coupling was activated and completed in 1M 11% N-Methylmorpholine (NMM) in DMF. Synthesised peptides and side chain protecting groups were cleaved from the resin using 95% trifluoroacetic acid (TFA), 2.5% triisopropylsilane (TIPS) and 2.5% water. The confirmation of the primary structure of the synthetic peptides and their purity was accomplished by reverse-phase HPLC and using MALDI-TOF and an LCQ-Fleet electrospray ion-trap mass spectrometer (Thermo Fisher Scientific, San Francisco, CA, USA).

### 3.4. RP-HPLC Analysis of the Two Novel Synthetic Peptides and Co-Elution Profiling of These Two Peptides with Their Respective Skin Secretion Counterparts

PS-Du and PS-Co were dissolved using 0.5% TFA/water solution to a concentration of 1 mg/L. They were respectively analysed by injecting 1200 µL synthetic peptide solution into the Waters RP-HPLC system using the same gradient as described in Section 3.2.

An additional 7 mg of each lyophilized skin secretion from *Phyllomedusa duellmani* and *Phyllomedusa coelestis,* were dissolved separately as described in Section 3.2. Afterwards, 200 µL of synthetic PS-Du and PS-Co solutions mentioned above were mixed with corresponding dissolved skin secretions. Both mixtures of 200 µL of peptide solution and 1000 µL of skin secretion solution were analysed using the Waters RP-HPLC system with the same gradient as described in Section 3.2.

### 3.5. Antimicrobial Activity Assays with the Two Novel Peptides and Their Structurally-Modified Analogues

Antimicrobial activity of each peptide was assessed by determination of minimal inhibitory concentrations (MICs), defined as the minimal concentration of antibiotic which inhibits growth following an overnight incubation with microorganisms. *Escherichia coli* (NCTC 10418) as a standard Gram-negative bacterium, *Staphylococcus aureus* (NCTC 10788) as a standard Gram-positive bacterium and *Candida albicans* (NCPF 1467) as a standard pathogenic yeast, were used in these experiments and were grown in Mueller-Hinton broth (MHB) for 18 h. Peptides were initially made as stock solutions using 1% dimethyl sulfoxide (DMSO) in phosphate-buffered saline (PBS), and the working solutions were prepared from these to achieve final concentrations of peptides from 512 to 1 mg/L. Peptide solutions were incubated with growth cultures (10^6^ colony forming units (CFU)/mL) in 96-well plates for 18 h at 37 °C. The growth of bacteria/yeast was detected by optical density (OD) measurements at a wavelength of 550 nm. The minimal inhibitory concentrations (MICs) of all four synthetic peptides were determined as the lowest concentration of peptide where no growth was detectable using an ELISA plate reader (Biolise BioTek EL808, Winooski, VT, USA).

### 3.6. Haemolysis Assay of the Two Novel Peptides and Their Modified Analogues

The haemolysis assay was performed using erythrocytes prepared from defibrinated horse blood (TCS Biosciences Ltd., Buckingham, UK). Two hundred µL of a 4% (*v*/*v*) suspension of erythrocytes in phosphate-buffered saline (PBS) were incubated with different concentrations of peptides prepared in PBS from 512 to 1 mg/L at 37 °C for 2 h. Lysis of erythrocytes was assessed by measurement of optical density at 550 nm using an ELISA plate reader (Biolise BioTek EL808, Winooski, VT, USA). Negative controls employed consisted of a 4% (*v*/*v*) erythrocyte suspension and PBS in equal volumes. Positive controls consisted of a 4% (*v*/*v*) erythrocyte suspension and an equal volume of 2% (*v*/*v*) of the non-ionic detergent, Triton X-100 (Sigma–Aldrich, St. Louis, MO, USA), in PBS solution.

### 3.7. Anti-Biofilm Activities of the Two Novel Peptides Tested on S. aureus Biofilm

The anti-biofilm activities of the two novel peptides were tested on *S. aureus* biofilm following a standard method as per the manufacturer’s instructions (Innovotech, Edmonton, Canada) for evaluating the MBEC (minimal biofilm eradication concentration). *S. aureus* (NCTC 10788) was incubated in MHB overnight at 37 °C and subcultured before being seeded onto the plate. 150 µL of 10^5^ CFU/mL bacteria culture were inoculated into each well of the MBEC assay plate (Innovotech). The biofilm was formed on the purpose-designed pegs after incubation for 48 h in an orbital-incubator at 37 °C, 150 rpm and appropriate humidity. Subsequently, a challenge plate was filled with 200 µL of peptide working solutions of each concentration in TSB and the lid with pegs was inserted into this plate after PBS washing steps. After incubation for 24 h at 37 °C, the pegs were washed using PBS again and then transferred into a recovery plate with 200 µL recovery solution (MHB/neutralising agents 20/0.5 (*v*/*v*)) in each well. A 30-min ultrasonic treatment was employed to disrupt the biofilm from the pegs and the recovery plate was incubated for another 24 h at 37 °C. The 96-well plate was analysed using an ELISA plate reader (Biolise BioTek EL808, Winooski, VT, USA) as described in the previous section. The minimal biofilm eradication concentration (MBEC) is defined as the lowest concentration where no growth of bacteria was detectable after biofilm was ultrasonically disrupted and further incubated in the recovery plate.

### 3.8. Bacterial Cell Membrane Permeability Assay of the Two Novel Peptides Using S. aureus

The membrane permeability assay was carried out using SYTOX Green Nucleic Acid Stain (Life technologies, Carlsbad, CA, USA) as descried by Roth et al [25]. Bacteria were incubated in Tryptic Soy Broth (TSB) (Sigma–Aldrich, St. Louis, MO, USA) at 37 °C overnight, after which 200 µL of bacterial culture was inoculated into 25 mL TSB and incubated at 37 °C for 3 h to achieve the logarithmic growth phase. Then, bacterial cells were harvested by centrifugation at 1000× *g* for 10 min at 4 °C, followed by two cell washing processes with 5% TSB in 0.85% NaCl solution. The washed bacterial cells were suspended in 5%TSB to achieve 1 × 10^8^ CFU/mL which was detectable at OD 590 nm = 0.7. Each well of the sample groups in a black 96 well plate (Fisher Scientific, Leicestershire, UK) contained a volume of 50 µL of bacterial suspension and 50 µL of peptide solution. Each well of the negative control group was constituted by a volume of 50 µL of bacterial suspension and 40 µL of 5% TSB. The positive control group was established by using 70% isopropanol-permeabilised bacterial cells, as described by Roth et al. [25], and was made by a volume of 50 µL of permeabilised bacterial cell suspension and 40 µL of 5% TSB. 10 µL of SYTOX green nucleic acid stain was added to each well to a final concentration of 5 µM. Meanwhile, the background fluorescence was measured using a volume of 90 µL 5% TSB and 10 µL SYTOX green nucleic acid stain at the same concentration. The black plate was incubated for 2 h at 37 °C in the dark. The fluorescent intensity of each well was recorded using an ELISA plate reader (Biolise BioTek EL808, Winooski, VT, USA) with excitation at 485 nm and emission at 528 nm.

### 3.9. Statistical Analysis

Data were subjected to statistical analysis using Prism (Version 5.0; GraphPad Software Inc., San Diego, CA, USA). Error bars in the graphs represent standard error of the mean (SEM) with experiments performed on more than three sets of replicates.

## 4. Discussion

Amphibian skin secretions have been proven to be a rich source of biomolecules for current lead drug discovery [26,27]. Frog skin peptides, as the predominant compounds in the secretions, exhibit a great potential for the treatment of many diseases in the areas of cancer and infection [8,28,29]. Phyllomedusine leaf frogs are one of the most remarkable subfamilies of amphibians and they have contributed much to the study of skin peptides in that they contain many varieties of unique bioactive peptides, such as phylloseptins, dermaseptins, medusins and phyllokinins [13,30,31]. Therefore, it is essential to continue investigations on the species of this subfamily to identify more novel peptides. So far, only 16 species have been studied from 59 species of this subfamily [12,32]. In this study, two virtually unstudied species of phyllomedusine frogs, *Phyllomedusa duellmani* and *Phyllomedusa coelestis,* were chosen. Unlike the well-studied species which are widely-distributed in South America, these two species are limited to remote areas of northern Peru with colonies only recorded in specific localities in mountainous areas. *Phyllomedusa duellmani* as an example, has an altitudinal range of 1850–1910 m above sea level [20]. These strict living environments may influence gene expression and contribute to unique skin defence peptide generation, some of which may show a great potential in drug lead discovery.

**Figure 13:**
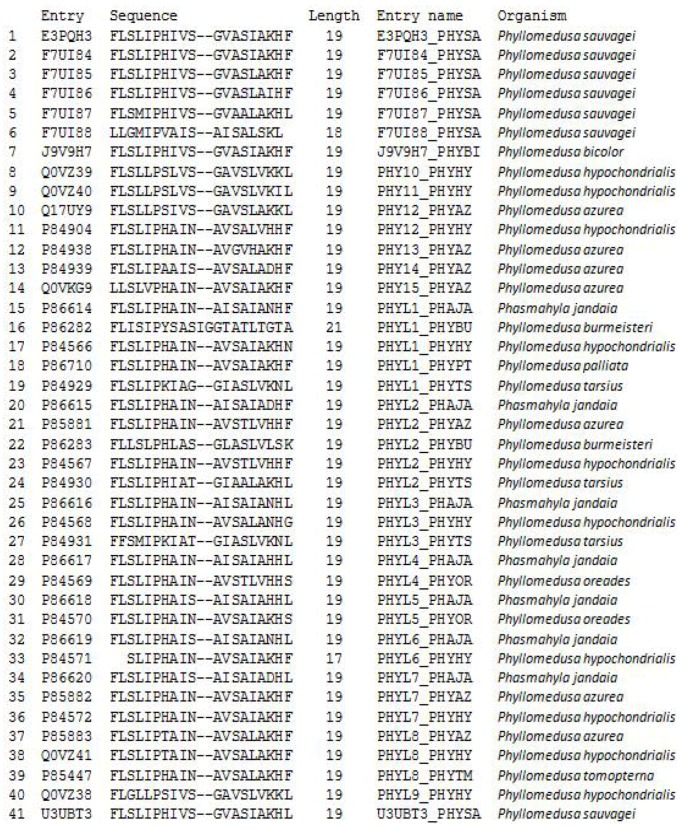
Phylloseptin sequences recorded in the Uniprot database.

In this study, two novel phylloseptin peptides, named PS-Du and PS-Co, from the skin secretions of *Phyllomedusa duellmani* and *Phyllomedusa coelestis,* respectively, were discovered. Since Leite reported the prototype phylloseptin in 2005, more than 40 novel phylloseptin (PS) peptides with highly-conserved amino acid sequences from the skin secretions of phyllomedusine leaf frogs, have been discovered [14,15,16,17,18,33]. According to the records in the Uniprot database (The Uniprot Consortium), PS peptides show some common characteristics with the presence of an n-terminal Phe residue, a Pro residue at position 6, a His residue at position 7 and a variable amidated *C*-terminal residue. Most of their primary structural characteristics are highly-conserved such as the n-terminal hexapeptide, FLSLIP- (Figure 13). Some phylloseptins possess different amino acids within this hexapeptide region with Leu and Ser residues replaced by Ile and Gly, respectively. This is a common phenomenon occurring in discrete peptide families among amphibian species. The different isoforms within peptide families provide useful phylogenetic information on the genetic mutations which have occurred during speciation, offering great benefits for the species to increase the survival capabilifrom cruel natural selection. Here, the encoded cDNAs isolated from *Phyllomedusa duellmani* and *Phyllomedusa coelestis,* proved to be highly-conserved.

Interestingly, such topological structural conservation is a very common phenomenon in other amphibian skin defence peptide families including dermaseptins, medusins and phyllokinins, from skin secretion of phyllomedusine leaf frogs [15,16,31]. These features have further revealed that they have developed a unique defence peptide expression approach from the precursors to the mature peptides.

PS-Co and PS-Du were extremely similar in structure with only two differences in amino acid sequence in position 2 and position 10. In addition, their secondary structures as predicted by SWISS-MODEL modeling software, showed a large proportion of α-helix from position 4 to position 18.

Interestingly, unlike one of the common features of most phylloseptin peptides, PS-Co and PS-Du both demonstrated a lysine (K) substitution for histidine (H) at position 7. This substitution increases the net positive charge of the peptide from His (pKa ~6.0, imidazole-nitrogen) to Lys (pKa ~10.5, ε-amino group), which might lead to enhancing antimicrobial activity given that it might improve electrostatic attraction and interaction with negatively-charged bacterial cell surfaces as well as conserving other structural parameters related to activity. The lysine substitution of histidine at position 7 in the two novel phylloseptins, might also indicate an additional evolutionary adaptation to aid the survival of these two species in a harsh environment [32]. To assess this prediction, we further synthesised two modified analogues with lysine substituted by histidine at position 7 in both peptides, naming these PS-Du K7H and PS-Co K7H, respectively. The synthetic K7H mutants of both peptides showed decreased antimicrobial activity but similar haemolytic activities. Compared with reported phylloseptins, these two novel phylloseptins exhibited more potent antimicrobial activities, especially against the Gram positive bacterium, S. aureus, to nearly the lowest MIC value of 8 mg/L. The anti-yeast activity of PS-Du and PS-Co with an MIC value of 16 mg/L, makes them the most effective among most reported phylloseptins [14,16,19,34]. Both peptides showed less potent inhibition of the Gram-negative bacterium E. coli (128 mg/L). As is known, Gram-negative bacteria have an extra outer cellular membrane and a large proportion of highly negatively-charged lipopolysaccharide (LPS) [35] making it more difficult for antimicrobial peptides to penetrate. Therefore, to disrupt the cell membrane, higher concentrations of these two novel phylloseptins were required to induce permeabilization of the inner membrane [34].

Meanwhile, both PS-Du and PS-Co exhibited potent anti-biofilm activity against a biofilm of *S. aureus*. Biofilm, containing a polysaccharide matrix, is a special product of many microorganisms for affording better protection and survival and can contribute to an increase in clinical infections caused by its resistance to many antibiotics [36]. Nosocomial infections have become a serious problem and 60% of these infections are associated with microorganism biofilm and most are Gram-positive bacteria-related infections [37,38,39]. It is thus essential to discover new efficient anti-biofilm drugs to treat biofilm-mediated infections. Antimicrobial peptides, which are natural defensive components of the innate immune system against microorganisms, are attractive candidates showing great potential in the treatment of bacterial biofilms [40,41,42]. Indeed, in previous studies, amphibian skin antimicrobial peptides have been demonstrated to eradicate *S. aureus* biofilm [17,43,44]. In this study, both PS-Co and PS-Du exhibited a potent action against *S. aureus* biofilm at a concentration of 16 mg/L, suggesting that these two phylloseptins resist bacterial biofilm. However, it is unclear as to how they passed through and disrupted the biofilm and thus further studies could combine cell staining and imaging systems for anti-biofilm mechanism investigations.

Currently, the mechanism of bacterial inhibition by antimicrobial peptides has been widely-accepted as non-specific membrane disruption, forming of toroidal pores and inducing damage to intact cell membranes increasing their permeability [45,46]. The membrane permeabilization assay employed here was a new approach to the study of the mechanism of these novel phylloseptins in inhibiting the growth of microorganisms. The interaction of peptides with *S. aureus* bacterial membranes was shown through cell membrane permeabilization enhancement with increasing peptide concentrations. However, PS-Du and PS-Co did not fully permeabilize cell membranes at their MIC of 8 mg/L. Regarding these lower concentrations of peptides, limited numbers or sizes of toroidal pores might be formed.

In summary, two novel phylloseptin peptides, named PS-Du and PS-Co, were identified in the skin secretion of *Phyllomedusa duellmani* and *Phyllomedusa coelestis,* respectively. Both peptides showed potent antimicrobial activity against a Gram-positive bacterium and a yeast and both were able to disrupt and eradicate *S. aureus* biofilm*,* in vitro. This study suggests that these phylloseptins may be promising candidates in the discovery and development of new antibiotic drugs and also provided new insights into natural antimicrobial drug design.

## Figures and Tables

**Table 1:** The minimal inhibitory concentrations (MICs) of PS-Du, PS-Co, PS-Du K7H and PS-Co K7H, against the three different microorganisms.

Peptide Name	Molecular Mass(Da)	MIC		
		*S. aureus*	*E. coli*	*C. albicans*
PS-Du	2049.5	8 mg/L	128 mg/L	16 mg/L
		(3.90 µM)	(62.45 µM)	(7.81 µM)
PS-Co	1971.5	8 mg/L	128 mg/L	16 mg/L
		(4.06 µM)	(64.93 µM)	(8.12 µM)
PS-Du K7H	2057.1	32 mg/L	512 mg/L	64 mg/L
		(15.56 µM)	(248.89 µM)	(31.12 µM)
PS-Co K7H	1979.1	32 mg/L	512 mg/L	64 mg/L
		(16.17 µM)	(258.70 µM)	(32.34 µM)
